# A Case of Strabismus in a Patient With Psoriasis

**DOI:** 10.1155/carm/2348907

**Published:** 2026-06-29

**Authors:** Karen Cravero, Emma Scott, Matthew Calestino, Nausheen Khuddus

**Affiliations:** ^1^ Transitional Year Residency Program, University of Central Florida, Gainesville, Florida, USA, ucf.edu; ^2^ Department of Research, HCA Florida North Florida Regional Hospital, Gainesville, Florida, USA; ^3^ Department of Research, University of Toledo, Toledo, Ohio, USA, utoledo.edu

**Keywords:** esotropia, psoriasis, psoriatic arthritis, strabismus

## Abstract

Psoriasis is a chronic immune‐mediated skin disease that may be associated with ocular manifestations, most commonly uveitis, keratoconjunctivitis, and blepharitis. Strabismus has been rarely reported in association with psoriasis. We present a woman with longstanding psoriasis, who developed acquired intermittent alternating esotropia with diplopia; she was later diagnosed with psoriatic arthritis. This case describes an uncommon ocular finding occurring with psoriasis and underscores the importance of coordinated, multidisciplinary care when new ocular or systemic symptoms arise.

## 1. Introduction

Psoriasis is a chronic immune‐mediated inflammatory skin disorder that affects approximately 2%–3% of the global population and is associated with a range of systemic manifestations. Ocular involvement has been increasingly recognized in patients with psoriasis, with reported conditions including uveitis, keratoconjunctivitis sicca, blepharitis, cataracts, glaucoma, punctate keratitis, and pinguecula [1–4]. These manifestations are typically related to inflammatory or infectious processes affecting ocular structures. Neurological ocular abnormalities, however, are rarely described in association with psoriasis.

Strabismus, defined as a misalignment of the eyes, most commonly arises from abnormalities in the extraocular muscles or their neurological control, rather than from systemic inflammatory disease. Although uncommon, inflammatory mechanisms have been implicated in certain forms of acquired strabismus, including Brown syndrome. Brown syndrome has been reported in association with systemic inflammatory disorders such as rheumatoid arthritis, Still’s disease, and systemic lupus erythematosus (SLE) [5, 6]. However, strabismus has rarely been reported in patients with psoriasis.

Psoriatic arthritis (PsA) is a chronic inflammatory arthropathy that develops in a subset of patients with psoriasis, with an estimated prevalence ranging from 0.05% to 0.25% in the general population. This increases to between 6% and 45% among individuals with psoriasis [1]. PsA most commonly manifests in the third or fourth decade of life and typically presents with asymmetric arthritis of the distal interphalangeal joints, dactylitis, enthesitis, and axial involvement.

Here, we report a patient with longstanding psoriasis, who developed acquired strabismus prior to the later diagnosis of PsA. To the best of the authors’ knowledge, only two prior cases of acquired strabismus associated with PsA have been reported [5, 7]. The purpose of this report is to describe this uncommon ocular finding in psoriasis and review ocular manifestations reported in this condition. Recognition of atypical ocular presentations may prompt earlier evaluation for associated systemic disease and improve clinical management.

## 2. Case Report

A Caucasian woman in her forties, with a longstanding history of psoriasis dating back to adolescence, presented to her ophthalmologist with complaints of dry eyes and difficulty focusing from a distance on a computer, despite corrective lenses. Her psoriasis had followed a relapsing‐remitting course with intermittent flares of cutaneous plaques managed with topical therapies. She had no prior diagnosis of PsA at the time of presentation. Her ocular history was notable for an episode of noninfectious anterior uveitis approximately 6 years prior, which resolved with corticosteroid therapy. At the time of strabismus onset, there was no evidence of active uveitis.

She reported new onset binocular diplopia and described having to close one eye to watch television comfortably. Comprehensive sensorimotor examinations demonstrated intermittent alternating esotropia. Fresnel prism lenses were prescribed. Magnetic resonance imaging (MRI) of the brain was performed to exclude intracranial or demyelinating pathology. This was unremarkable.

Despite prism correction, diplopia progressively worsened over the following years. Approximately 3 years after the onset of strabismus, the patient developed bilateral heel pain and lower back pain. Serologic evaluation including antinuclear antibodies (ANA), HLA‐B27, and rheumatoid factor were negative. Imaging studies revealed bilateral heel enthesitis and lumbar enthesitis. With characteristic musculoskeletal findings and exclusion of alternative etiologies, a diagnosis of PsA was established. These tests were crucial in differentiating PsA from other possible diagnoses and illustrate the importance of a targeted diagnostic approach when presented with a patient who exhibits both articular and dermatological symptoms. She was subsequently initiated on secukinumab for the management of PsA and later underwent surgical correction of her strabismus.

## 3. Discussion

Psoriasis is a chronic immune‐mediated inflammatory disorder characterized by red, scaly patches, affecting approximately 1%–3% of the global population [1]. Psoriasis has well‐documented extracutaneous manifestations, including ocular involvement. The most frequently reported ophthalmologic findings in patients with psoriasis include keratoconjunctivitis sicca, blepharitis, chronic conjunctivitis, and uveitis. Ocular surface disease and uveitis represent the most common inflammatory presentations. In contrast, disorders of ocular motility such as strabismus are rarely described in association with psoriasis.

Strabismus typically results from neuromuscular imbalance, mechanical restriction, or neurologic dysfunction, rather than systemic inflammatory skin disease [8]. Isolated reports have documented acquired Brown syndrome and other forms of strabismus in patients with inflammatory conditions, such as psoriasis and PsA [5]. However, the mechanism underlying this association remains unclear. Proposed explanations include localized inflammatory involvement of extraocular muscles or surrounding connective tissue, though definitive pathophysiologic links have not been established.

In this case, strabismus developed years after the initial diagnosis of psoriasis and preceded the eventual diagnosis of PsA by approximately 3 years. While a temporal association is observed, causality cannot be established. It remains uncertain whether the ocular motility disturbance represented an inflammatory manifestation related to psoriasis or an unrelated coincidental finding.

Among patients with psoriasis, a significant proportion, ranging from 6% to 41%, may eventually develop PsA, an inflammatory arthritis affecting peripheral and axial joints [2, 9]. This prevalence underscores the possible connection between these two conditions and emphasizes the progressive nature of the disease. This course often demonstrates patients enduring psoriasis for many years before development of arthritis [1, 2]. The pathogenesis of PsA involves immune mediated pathways driven by T‐cell activation and cytokines, such as tumor necrosis factor‐alpha (TNF‐alpha) and interleukin‐17 (IL‐17), which promote joint inflammation and may also impact other tissues, including ocular structures [10, 11]. The multifaceted pathology of PsA includes an interplay between genetic predispositions and environmental triggers, which together lead to the immune‐mediated inflammatory processes affecting various body systems.

Eye involvement has been reported in 10%–12% of patients with psoriasis. A wide range of ocular manifestations may occur, including keratoconjunctivitis sicca, blepharitis, chronic conjunctivitis, uveitis, and corneal lesions [12]. PsA shares certain ocular manifestations with other systemic inflammatory diseases such as rheumatoid arthritis, thyroid disease, and SLE [9, 13]. However, unique presentations, such as strabismus, are rare and may reflect underrecognized ocular involvement in psoriasis or broader inflammatory mechanisms, rather than a defining feature of PsA [14].

Although uncommon, reports linking psoriasis and PsA with strabismus have been documented in the literature, dating back several decades [15, 16]. For instance, a study in Ireland found that about 15% of children with psoriasis had strabismus [16]. Thorne et al. reported a case of acquired Brown syndrome, a subtype of strabismus involving the superior oblique muscle, in a 42‐year‐old woman with psoriasis [7], while the same condition was reported in a 24‐year‐old woman with PsA [5]. These limited cases suggest specific mechanistic involvement that merits further investigation [6, 7, 14, 15].

Beyond localized ocular findings, psoriasis is recognized as a systemic inflammatory condition associated with comorbidities such as metabolic syndrome, cardiovascular disease, and other immune‐mediated disorders [17]. Ocular manifestations may reflect broader inflammatory involvement rather than isolated ophthalmic pathology. Although a direct relationship between strabismus and specific psoriasis‐related comorbidities has not been clearly established, the presence of atypical ocular symptoms warrants careful multidisciplinary evaluation and longitudinal monitoring.

This case adds to the limited literature describing acquired strabismus in patients with psoriasis. It highlights the importance of recognizing uncommon ocular presentations during the disease course. Further study is needed to clarify whether such findings represent inflammatory sequelae or incidental coexistence.

## Funding

No funding was received for this research.

## Conflicts of Interest

The authors declare no conflicts of interest.

## Data Availability

Data sharing is not applicable to this article as no datasets were generated or analyzed during the current study.
